# Solubilization Behavior of Homopolymer in Its Blend with the Block Copolymer Displaying the Feature of Lower Critical Ordering Transition

**DOI:** 10.3390/polym13193415

**Published:** 2021-10-05

**Authors:** Yu-Hsuan Lin, Chang-Cheng Shiu, Tien-Lin Chen, Hsin-Lung Chen, Jing-Cherng Tsai

**Affiliations:** 1 Department of Chemical Engineering, National Tsing Hua University, Hsinchu 30013, Taiwan; acheda0120@gmail.com (Y.-H.L.); dijeng9@yahoo.com.tw (C.-C.S.); 2 Department of Chemical Engineering, National Chung Cheng University, Chia-Yi 62102, Taiwan; gemey24@gmail.com (T.-L.C.); chmjct@ccu.edu.tw (J.-C.T.)

**Keywords:** block copolymer, homopolymer, blend, lower critical ordering transition, interdomain distance

## Abstract

Blending with homopolymer offers a facile approach for tuning the microdomain morphology of block copolymer, provided that the homopolymer chains are uniformly solubilized in the corresponding microdomain to swell the junction point separation. Here we studied the solubilization behavior of poly(4-vinyl pyridine) homopolymer (h-P4VP) in the lamellar microdomain formed by its blends with a poly(ethylene oxide)-block-poly(4-vinyl pyridine) (PEO-*b*-P4VP) showing the feature of lower critical ordering transition (LCOT) in terms of weaker segregation strength at lower temperature. We revealed that, while the conventional criterion of homopolymer-to-block molecular weight ratio for attaining uniform solubilization was applicable to LCOT blend, there was an excess swelling of junction point separation upon the addition of homopolymer, leading to a decrease of interdomain distance with increasing homopolymer composition. This anomalous phenomenon was attributed to the reduction of interfacial free energy due to the incorporation of P4VP homopolymer into the microdomain interface.

## 1. Introduction

Microphase separation of the diblock copolymer, A-*b*-B, can generate a variety of long-range ordered microdomains, including one-dimensionally stacked lamellae (*LAM*), hexagonally packed cylinders (*HEX*), double gyroids and body-centered cubic (BCC)-packed spheres [1,2]. The type of structure formed is governed by the interfacial free energy and the conformational free energy associated with the stretching of the block chains [3,4]. These effects are parameterized into the segregation strength expressed by *χN* (with *χ* and *N* being the Flory–Huggins interaction parameter and the overall degree of polymerization of the copolymer, respectively) and the constituent volume fraction for constructing the universal phase diagram [3,4]. At sufficiently high compositional asymmetry, the microdomain interface becomes hyperbolically curved to alleviate the stretching of the majority blocks, leading to the formation of cylindrical or spherical domains by the minority blocks.

Another way to tune the microdomain morphology of diblock copolymer is through blending A-*b*-B with the corresponding homopolymer, h-A, to form a thermodynamically single-phase mixture exhibiting only microphase separation [2,5,6,7,8]. When the h-A chains are allowed to enter microdomain A and mix uniformly with A blocks (called “uniform solubilization”), the nearest-neighbor distance between the junction points (called “junction point separation”) locating in the interface will be swollen to alleviate the stretching of A blocks and h-A chains [5,6]. The increase of junction point separation increases the interfacial free energy, and B block chains will be compressed along the domain interface to maintain their normal segmental density. Once the entropic penalty associated with such a chain compression is too large, a morphological transformation from microdomain with lower curvature (e.g., lamellae) to the one with higher curvature (e.g., cylinder) will take place to relieve the conformational entropy loss. 

Consequently, an essential condition for achieving domain morphology transformation via homopolymer blending is the uniform solubilization of h-A in microdomain A to form the so-called “wet-brush” mixture of A blocks and h-A chains [5,6,7]. Achieving such a condition is not trivial due to the need to overcome the free energy penalty associated with the perturbation of conformational free energy as well as the increase of interfacial free energy. It has been established that wet-brush blending can be attained when the molecular weight of h-A (*M*_h-A_) is smaller than that of A block (*M*_b-A_), i.e., *r* = *M*_h-A_/*M*_b-A_ < 1 [5,6,7,8,9,10,11,12]. If h-A and block A have approximately the same molecular weight (i.e., *r* ~ 1), h-A chains may still enter microdomain A, but they tend to be segregated into the middle region of the domain, forming the “dry-brush” mixture with A blocks [5,6,13]. Dry-brush type of blending is unable to induce transformation of microdomain morphology, since it only causes a continuous swelling of the size of domain A without altering the junction point separation and the local interfacial curvature [5]. 

It should be noted that the molecular weight criteria for homopolymer solubilization were established using the diblock copolymer system (e.g., polystyrene-*block*-polyisoprene (PS-*b*-PI)) showing a reduction of segregation strength with increasing temperature [5,6]. This type of diblock system is said to exhibit the “upper critical ordering transition (UCOT)”, where the order–disorder transition (ODT) occurred on heating. The free energy components of the copolymer blends displaying UCOT behavior compose the interfacial free energy, the conformational free energy of block and homopolymer chains, the free energy of mixing of h-A and A block, and the free energy associated with the translational entropy the junction points [5]. Moreover, this type of system is considered to follow the melt incompressibility condition, where the sum of the local number densities of A and B segments is constant when they mix in the disordered melt or in the microdomain interface [1,3,4]. 

There is another class of diblock copolymer showing opposite temperature dependence of segregation strength, namely, the net repulsion between A and B segments becomes stronger at higher temperature, such that the ODT may take place on cooling. The copolymer is then said to display the “lower critical ordering transition (LCOT)” [14,15,16,17,18,19]. LCOT of diblock copolymer was first identified by Russell et al. [14]. and its origin was attributed to the disparity in the thermal expansivities of the constituent blocks, which is identical to that of the homopolymer blends exhibiting lower critical solution temperature (LCST) phase diagram [14,20,21]. In this case, the constraint of the free volume of the more expansive component by the less expansive one results in an entropy loss when they mix intimately with each other. As a result, a repulsive force with entropic in origin develops to trigger the microphase separation at elevated temperature. The equation of state theories that take account of the melt compressibility and the volume of mixing provide the appropriate theoretical framework for predicting the LCOT phase behavior [20,21,22,23]. 

Considering the more complex interplay among the free energy components, it is of fundamental significance to examine if the blend of a diblock copolymer showing the feature of LCOT behavior with its corresponding homopolymer exhibits different phase structure from that of the conventional UCOT blend. The present work sheds light on the solubilization behavior of homopolymer in such a blend system through studying the blends of poly(4-vinyl pyridine) homopolymers (h-P4VP) with different molecular weights and a lamellae-forming poly(ethylene oxide)-*block*-poly(4-vinyl pyridine) (PEO-*b*-P4VP) showing the signature of LCOT behavior in terms of weaker segregation at lower temperature. It will be shown that, while the wet-brush criterion established for the conventional UCOT blend was still applicable to the LCOT system, the LCOT blend showed a higher degree of swelling of the junction point separation upon the addition of homopolymer, even resulting in a decrease of interdomain distance with increasing h-P4VP composition. The excess swelling of junction point separation will be discussed in connection with the reduction of the interfacial free energy upon incorporating h-P4VP segments into the microdomain interface. The excess swelling of junction point separation constitutes another anomalous feature of LCOT block copolymer, besides the opposite temperature variation of segregation strength, compared with the UCOT system.

## 2. Materials and Methods

### 2.1. Materials

PEO-*b*-P4VP with the number average molecular weights of PEO and P4VP blocks of 5000 g/mol and 7200 g/mol, respectively, was purchased from Polymer Source Inc. (Montreal, QC, Canada) This sample was denoted as EO4VP. A series of P4VP homopolymers with different molecular weights were also acquired from Polymer Source Inc. Table 1 tabulates the molecular characteristics of the copolymer and homopolymer samples used here. The h-P4VP samples with the number average molecular weights of 1000, 1700, 2300, and 4300 g/mol are denoted as h-P4VP1, h-P4VP2, h-P4VP3, and h-P4VP4, respectively. Chloroform purchased from TEDIA Inc. (Fairfield, OH, USA.) was used as the solvent for preparing the blends.

### 2.2. Methods

#### 2.2.1. Sample Preparation

In the case of neat EO4VP, the copolymer sample was dissolved in chloroform to obtain a homogeneous solution by stirring at 40 °C for 1 hour. The homogeneous solution was poured onto the petri dish followed by evaporating most solvent at room temperature for 48 h to form the as-cast film. The film was subsequently dried in vacuum at 40 °C for 72 h to remove the residual solvent.

For the preparation of the blends of EO4VP and h-P4VP, the two components were dissolved in chloroform according to the desired blend compositions. The solutions were then poured onto the petri dish followed by drying at the same condition as that used for preparing the neat copolymer film. Table S1 of the Supporting Information lists the compositions of the blend samples prepared here, where *f_P4VP_* denotes the overall volume fraction of P4VP in the blend and *Φ_EO4VP_* signifies the volume fraction of the diblock copolymer in the blend. 

#### 2.2.2. Small Angle X-ray Scattering (SAXS) Measurement

Temperature-dependent SAXS experiments were conducted with a Bruker N8 Horizon SAXS instrument (Karlsruhe, Germany) in a cooling cycle to investigate the structures of the blends at different temperatures. The SAXS instrument was furnished with a IμS micro-focus X-ray generator operated at 50 kV × 1000 µA. The wavelength of the X-ray source λ was 0.154 nm. Data were collected using a VANTEC-500 area detector with 2048 × 2048 pixel resolution located at 663.6 mm from the sample producing a *q* range of 0.1–3.5 nm^-1^, where *q* = 4πsin(θ/2)/λ with θ being the scattering angle. The collected scattering patterns were radially averaged to obtain one-dimensional scattering intensity profiles. All the scattering profiles were corrected for the scatterings from air and cell. For the temperature-dependent measurement, the samples were first heated to 200 °C followed by stepwise cooling to collect the SAXS profiles in situ. The samples were equilibrated at each temperature for 30 min followed by data acquisition for 5 min. The measuring temperatures all situated above the crystallization temperature (<40 °C, as found in the DSC thermograms) of the PEO block to avoid the disturbance of crystallization. 

## 3. Results and Discussion

**Figure 1:**
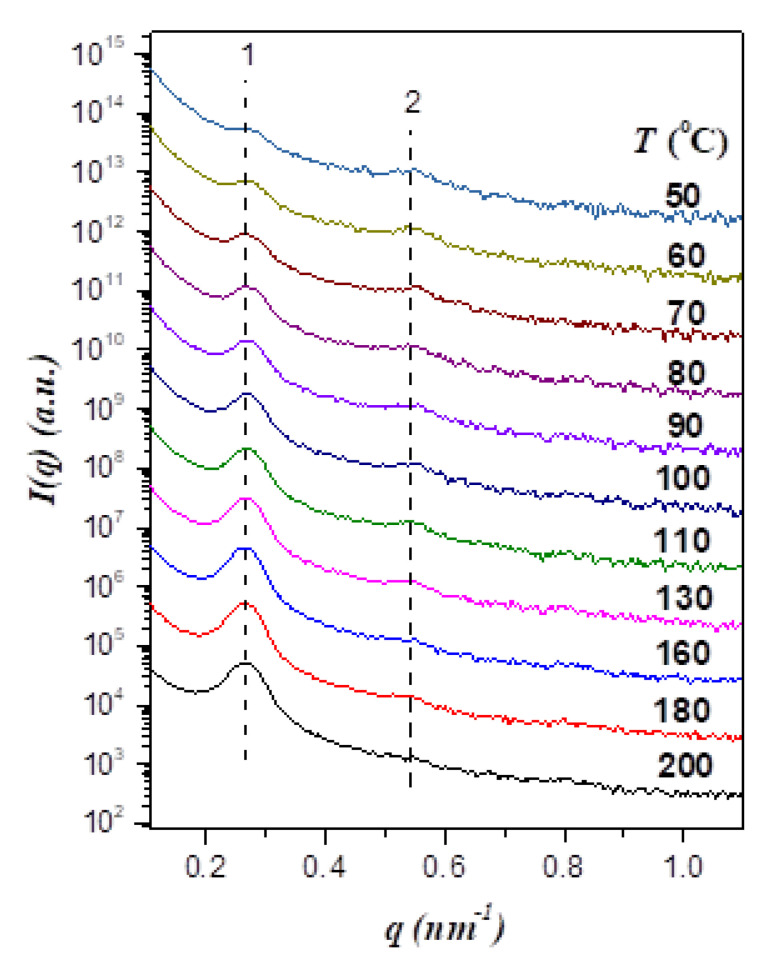
Temperature-dependent SAXS profiles of neat EO4VP collected in a cooling cycle. The scattering curves are shifted vertically for the clarity of presentation. The SAXS profiles demonstrate the formation of lamellar morphology over the temperature range studied. The primary peak diminished and broadened progressively with decreasing temperature.

Our previous study demonstrated that poly(ethylene oxide)-*block*-poly(2-vinyl pyridine) (PEO-*b*-P2VP) displayed LCOT phase diagram [18]. As the chemical analogue of PEO-*b*-P2VP, PEO-*b*-P4VP would be expected to show similar phase behavior. Figure 1 displays the temperature-dependent SAXS profiles of neat EO4VP, where the sample was first heated to 200 °C to erase the previous solvent history followed by collecting the scattering profiles in a cooling cycle. The SAXS curve at 200 °C displayed 2 peaks with the position ratio of 1:2, indicating that the copolymer formed a lamellar morphology with the interdomain distance (*D*) of 23.4 nm. The intensity of the primary peak was found to drop progressively with decreasing temperature, which seemed to suggest a reduction of segregation strength in the cooling process. Nevertheless, the difference in electron density between PEO and P4VP was found to decrease with decreasing temperature because of the disparity in their thermal expansion coefficients, as demonstrated in Figure S1 of the Supporting Information. Since the reduction of electron density contrast between PEO and P4VP domains could also cause the diminishment of peak intensity, identifying the phase behavior of EO4VP based solely on the temperature variation of scattering intensity was not unambiguous. 

**Figure 2:**
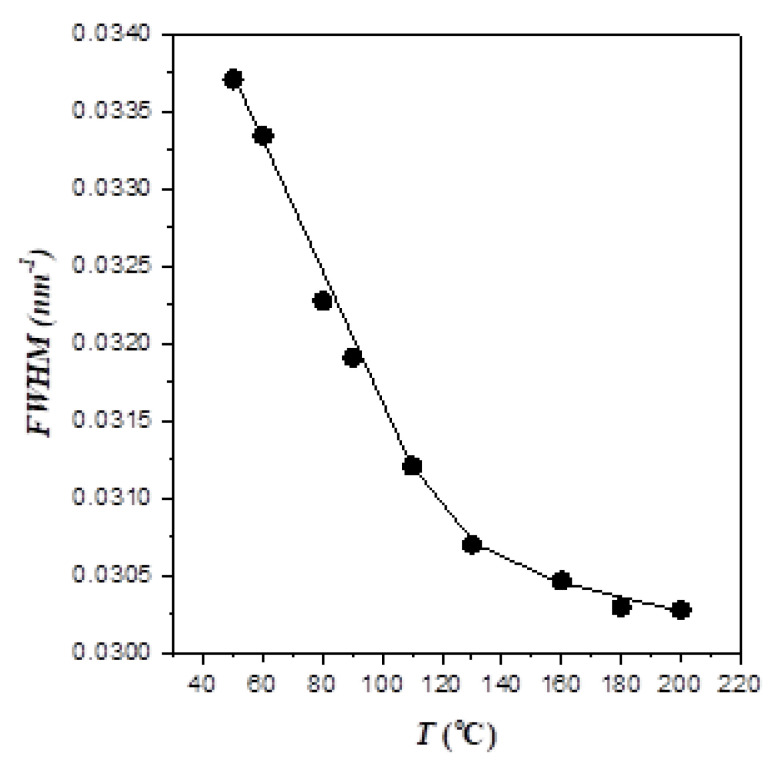
Full width at half-maximum (*FWHM*) of the primary scattering peak as a function of temperature for neat EO4VP. The peak width increased with decreasing temperature, signaling a decrease of segregation strength with decreasing temperature.

The effect of temperature on the segregation strength was alternatively elucidated from the change of the primary peak width in the cooling process, as demonstrated in Figure 2 showing the width at the half-height of this peak as a function of temperature. It can be seen that the peak broadened progressively with decreasing temperature, signaling a reduction of segregation strength with decreasing temperature. Moreover, we found that the blend of another symmetric PEO-*b*-P4VP (*M*_b-PEO_ = 4000 g/mol; *M*_b-P4VP_ = 6000 g/mol) and h-P4VP1 with f_P4VP_ = 0.68 exhibited an order–order transition from *LAM* to *HEX* phase on cooling (see Figure S2 of the Supporting Information), which was in the opposite direction to that displayed by the conventional UCOT system. These experimental observations attested that the segregation strength of EO4VP became weaker at a lower temperature. That is, this copolymer system displayed the feature of LCOT behavior, though the disordered state was not accessible before the occurrence of the crystallization of PEO block below its melting point.

The morphology and phase behavior of EO4VP/h-P4VP blends were also probed by SAXS. Here we used EO4VP/h-P4VP1 blend with the lowest molecular weight ratio *r* = *M*_h-P4VP_/*M*_b-P4VP_ = 0.14 as the representative to demonstrate the LCOT characteristic of the blends, since the temperature-dependent SAXS results of the other blends basically showed the same features. 

**Figure 3:**
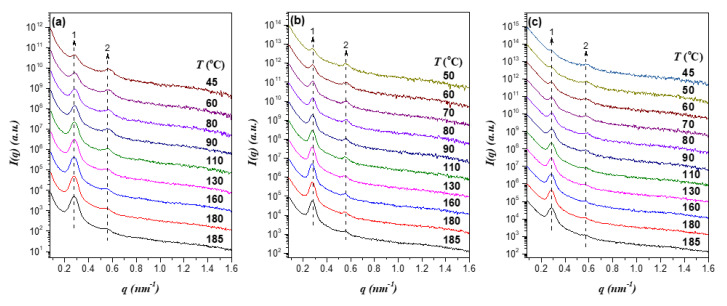
Temperature-dependent SAXS profiles of the EO4VP/h-P4VP1 blends with the overall volume fractions of P4VP of (**a**) *f_P4VP_* = 0.66, (**b**) *f_P4VP_* = 0.69, and (**c**) *f_P4VP_* = 0.71 collected in a cooling cycle. The scattering curves are shifted vertically for the clarity of presentation. As that found for neat EO4VP, the primary peak diminished and broadened progressively with decreasing temperature.

Figure 3 presents the temperature-dependent SAXS profiles of the EO4VP/h-P4VP1 blends with the overall volume fraction of P4VP, *f_P4VP_*, ranging from 0.66 to 0.71. The SAXS curves of all samples displayed two peaks with integral position ratio, showing that the blends also formed lamellar morphology over the temperature range studied. Similar to neat EO4VP, the primary peak diminished and broadened with decreasing temperature; consequently, EO4VP/h-P4VP1 blends also exhibited the feature of LCOT type of phase behavior.

**Figure 4:**
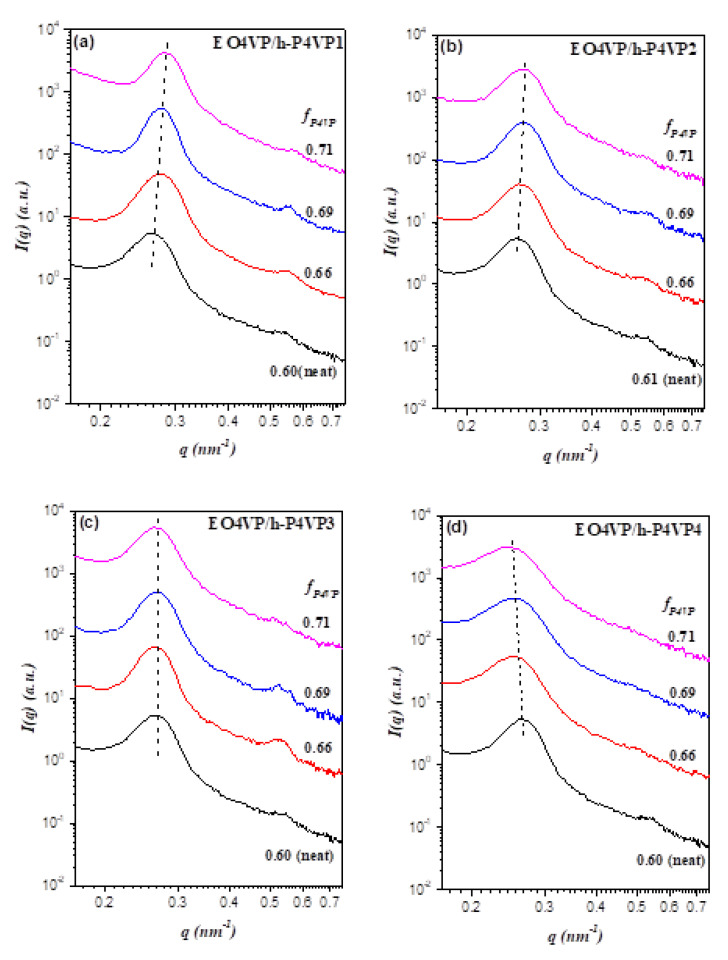
Composition-dependent SAXS profiles at 180 °C of the blends of EO4VP with (**a**) h-P4VP1, (**b**) h-P4VP2, (**c**) h-P4VP3, and (**d**) h-P4VP4. The dashed lines are drawn to illustrate the effect of composition on primary peak position. The primary peak shifted to higher *q* with increasing *f_P4VP_* for the blends with h-P4VP1 and h-P4VP2, while the peak position remained largely unperturbed for h-P4VP3 blend. The peak of h-P4VP blend shifted to lower *q* with increasing *f_P4VP_*.

Over the range of h-P4VP molecular weight studied, all blends were found to show weaker segregation strength at lower temperature; nevertheless, the perturbations of interdomain distance and junction point separation upon incorporating h-P4VP into the P4VP lamellar microdomain depended on h-P4VP molecular weight. Figure 4a shows the SAXS profiles of EO4VP/h-P4VP1 blends (*r* = 0.14) with various compositions at a representative temperature, 180 °C. The scattering peaks associated with the lamellar structure were found to shift to higher *q* with increasing *f_P4VP_*, indicating that the interdomain distance *D* became smaller with the addition of more h-P4VP. This was rather unexpected, as the previous studies of diblock copolymer blends had predominantly observed the swelling of interdomain distance once the homopolymer was solubilized in the corresponding microdomain [5,6]. 

The composition-dependent SAXS profiles of EO4VP/h-P4VP2 blends (*r* = 0.24) displayed in Figure 4b showed the formation of lamellar morphology; the scattering peaks were again found to shift towards higher *q* with increasing *f_P4VP_*, but the shift was less pronounced compared to that displayed by EO4VP/h-P4VP1 blend. The SAXS curves of EO4VP/h-P4VP3 blends (*r* = 0.23) shown in Figure 4c demonstrated that, while the blends still formed lamellar structure, the positions of the scattering peaks appeared to be fixed, implying that the interdomain distance remained largely unperturbed upon blending with the homopolymer. For the blend with h-P4VP4 bearing the highest molecular weight (*r* = 0.60) among the h-P4VP samples used, the SAXS profiles in Figure 4d revealed the shift of scattering peaks to lower *q* with the increase of P4VP composition. In this case, blending with h-P4VP tended to swell the interdomain distance, which was in clear contrast to the composition dependence of *D* displayed by the other three blends. 

**Figure 5:**
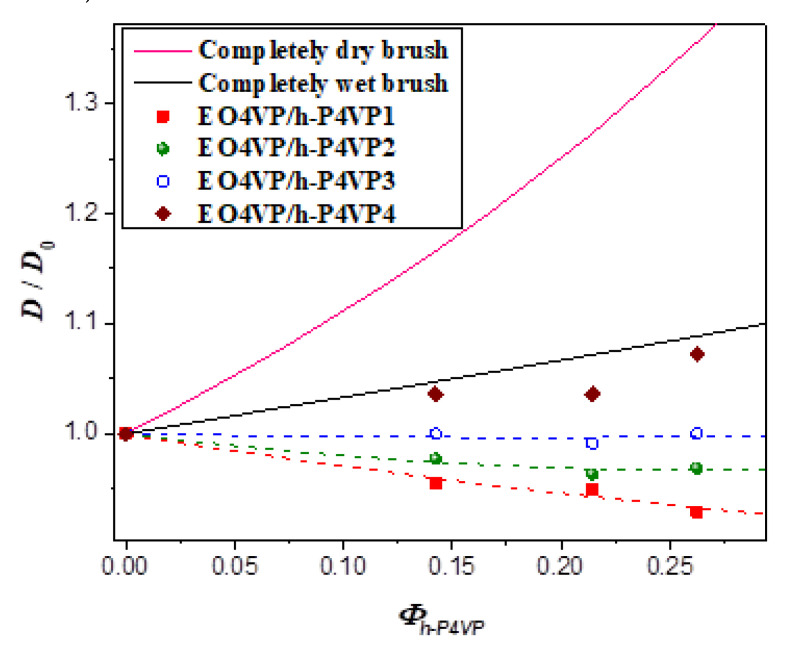
The normalized interdomain distance *D*/*D*_0_ as a function of the overall volume fraction of h-P4VP *Φ_h-P4VP_* of the blends at 180 °C. The solid curves represent the normalized interdomain distances calculated for the scenarios of dry-brush and wet-brush behavior with the assumption of unperturbed surface free energy.

Figure 5 plots the interlamellar distance of the blend normalized by that of neat EO4VP (*D*_0_), *D*/*D*_0_, as a function of the total volume fraction of h-P4VP, *Φ_h-P4VP_* = 1 − *Φ_EO4VP_*, in the blends at 180 °C. The composition dependence of *D*/*D*_0_ was seen to depend on the molecular weight of h-P4VP. For the blend with h-P4VP1 bearing the lowest molecular weight (*r* = 0.14), the interdomain distance decreased with increasing h-P4VP content, implying that the junction point separation was swollen significantly by h-P4VP, making the lamellar thickness even smaller than that in neat EO4VP. The interdomain distance of the blend with h-P4VP2 (*r* = 0.24) also showed similar composition dependence, but the drop of *D*/*D*_0_ was less pronounced. The interdomain distance remained virtually constant for h-P4VP3 blend (*r* = 0.32), whereas it increased monotonically with increasing homopolymer composition when the molecular weight of h-P4VP was increased to 4300 g/mol (*r* = 0.60). 

The reduction of interdomain distance with increasing homopolymer composition observed for the blends with h-P4VP1 and h-P4VP2 offered clear-cut evidence of wet-brush mixing between P4VP blocks and h-P4VP. It was, however, unclear if h-P4VP4 blend displayed wet-brush or dry-brush behavior because both may lead to swelling of interdomain distance. This problem was resolved by calculating the junction point separation *a_j_* in the blend normalized by that in the neat copolymer, *a_j_*/*a*_*j*0_ via [5]

(1) $$\frac{a_{j}}{a_{j0}}={\left[\frac{D}{D_{0}}\times \left(1-\Phi_{h-P4VP}\right)\right]}^{-0.5}$$

Equation (1) assumes that all added homopolymer was solubilized into the microdomain. This was a reasonable assumption for the blend systems studied here, in that no sign of macrophase separation was observed.

**Figure 6:**
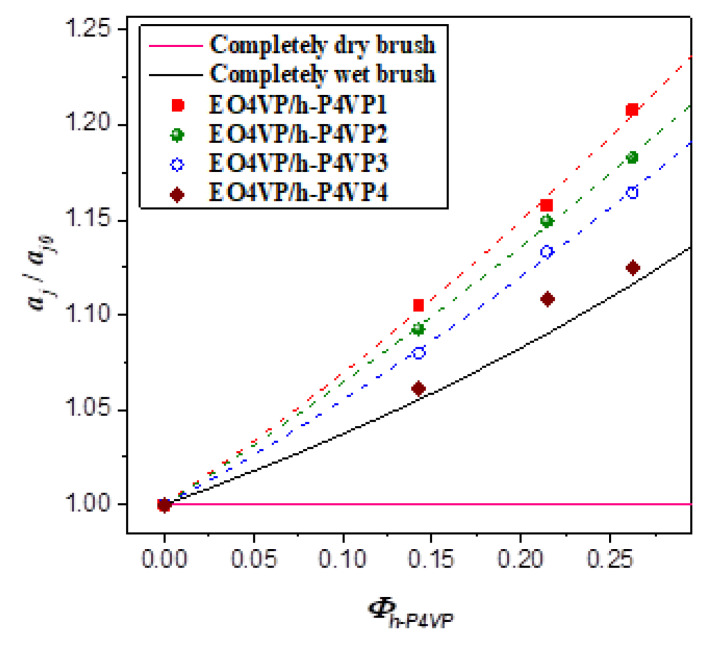
The normalized junction point separation *a_j_*/*a*_*j*0_ as a function of the overall volume fraction of h-P4VP *Φ_h-P4VP_* of the blends at 180 °C. The solid curves represent the normalized junction point separations calculated for the scenarios of dry-brush and wet-brush behavior with the assumption of unperturbed surface free energy.

Figure 6 shows the calculated *a_j_*/*a*_*j*0_ as a function of *Φ_h-P4VP_*. For the four blend systems studied, the values of *a_j_*/*a*_*j*0_ situated above 1.0 (*a_j_*/*a*_*j*0_ = 1 for dry-brush mixing) and increased monotonically with increasing h-P4VP composition, indicating that they all exhibited wet-brush behavior, where h-P4VP was uniformly solubilized in P4VP microdomains. Since the values of *r* associated with the four blends all fell below 1.0, the results suggested that the classical criterion of molecular weight ratio for wet-brush behavior, i.e., *r* < 1, was applicable to the blends showing the feature of LCOT behavior.

Although all h-P4VP samples formed wet-brush mixture with P4VP blocks, the degree of swelling of the junction point separation was found to decrease with increasing h-P4VP molecular weight. If the added homopolymer was uniformly solubilized in P4VP microdomain, swelling of the junction point separation would alleviate the stretching of block and homopolymer chains; however, this effect was counteracted by the increase of interfacial free energy arising from the enlargement of interfacial area. The fact that *a_j_*/*a*_*j*0_ was larger in the blend implied that the corresponding interfacial free energy per unit area (i.e., the surface free energy) of the lamellar phase was lower. In other words, the surface free energy was modified by the presence of h-P4VP in a molecular weight-dependent manner. 

Here, we further compared the experimentally observed *D*/*D*_0_ and *a_j_*/*a*_*j*0_ with the values expected for completely dry brush and completely wet brush behavior. Because the thickness of PEO microdomain was unperturbed in dry-brush blend, the expected values are *a_j_*/*a*_*j*0_ = 1 and *D*/*D*_0_ = (1 − *f_P4VP_*^0^)/(1 − *f_P4VP_*) with *f_P4VP_*^0^ being the volume fraction of P4VP in neat EO4VP. The calculations of *a_j_*/*a*_*j*0_ and *D*/*D*_0_ for wet-brush behavior were not straightforward. Here we adopted the model developed by Tanaka et al., originally derived for the ternary blend of a diblock copolymer with the two corresponding homopolymers [5], to obtain the formula of *a_j_*/*a*_*j*0_ and *D*/*D*_0_ of wet-brush blend.

For the binary mixture of A-*b*-B and h-A forming the lamellar morphology, the free energy per chain *F_chain_* is given by [5]

(2) $$\frac{F_{chain}}{k_{B}T}=\gamma \sum +\frac{3}{2}\left(\frac{D_{A}{}^{2}}{N_{A}a^{2}}+\frac{D_{B}{}^{2}}{N_{B}a^{2}}\right)+\frac{N_{A}}{2}\left[\frac{1-\phi_{h-A}}{N_{A}}\ln\left(1-\phi_{A}\right)+\frac{\phi_{h-A}}{P_{A}}\ln\phi_{h-A}\right]$$

where *D_i_*, *a*, *N_i_*, *P_A_*, *Σ*, *γ* and *ϕ_h-A_* are thickness of microdomain *i* (*i* = A or B), Kuhn length (assuming the same for A and B blocks), degree of polymerization (DP) of block *i*, DP of homopolymer A uniformly solubilized in its microdomain, the cross-sectional area per junction point, the surface free energy, and the volume fraction of homopolymer A in microdomain A, respectively. The three terms at the right-hand side of Equation (2) correspond to the interfacial free energy, the conformational free energy of the block chains and the free energy of mixing of h-A and A block in microdomain A, respectively.

Neglecting the volume of mixing that might exist in the interfacial region in which A and B segments mix with one another, the volume of one block chain is expressed in terms of the cross-sectional area of the junction point as

(3) $$N_{A}a^{3}=\sum D_{A}\left(1-\phi_{h-A}\right)$$

(4) $$N_{B}a^{3}=\sum D_{B}$$

Substituting Equation (3) and Equation (4) into Equation (2) yields

(5) $$\frac{F_{chain}}{k_{B}T}=\gamma \sum +\frac{3}{2}\left[\frac{N_{A}a^{4}}{{\left(1-\phi_{h-A}\right)}^{2}\sum^{2}}+\frac{N_{B}a^{4}}{\sum^{2}}\right]+C$$

where *C* is a constant. The equilibrium cross-sectional area of the blend, *Σ_eq_*, is obtained by minimizing the total free energy to yield

(6) $$\sum_{eq}={\left(\frac{3a^{4}}{\gamma }\right)}^{\frac{1}{3}}{\left[\frac{N_{A}}{{\left(1-\phi_{h-A}\right)}^{2}}+N_{B}\right]}^{\frac{1}{3}}$$

The cross-sectional area per junction point in neat block is given by

(7) $$\sum_{eq0}={\left(\frac{3a^{4}}{\gamma_{0}}\right)}^{\frac{1}{3}}{\left(N_{A}+N_{B}\right)}^{\frac{1}{3}}$$

where γ_0_ is the surface free energy of the lamellar microdomain in neat diblock copolymer. Combining Equation (6) and Equation (7), the normalized junction point separation is given by

(8) $$\frac{a_{j}}{a_{j0}}={\left(\frac{\sum_{eq}}{\sum_{eq0}}\right)}^{\frac{1}{2}}={\left(\frac{\gamma_{0}}{\gamma }\right)}^{\frac{1}{6}}{\left[\frac{f_{A}}{{\left(1-\phi_{h-A}\right)}^{2}}+f_{B}\right]}^{\frac{1}{6}}$$

where *f_i_* is the overall volume fraction of component *i* in the blend. The corresponding normalized interdomain distance is obtained as

(9) $$\frac{D}{D_{0}}={\left(\frac{\gamma }{\gamma_{0}}\right)}^{\frac{1}{3}}\left(\frac{f_{A}}{1-\phi_{h-A}}+f_{B}\right)\frac{1}{{\left[\frac{f_{A}}{{\left(1-\phi_{h-A}\right)}^{2}}+f_{B}\right]}^{\frac{1}{3}}}$$

Figure 5 shows the comparison of the experimentally observed *D*/*D*_0_ with the values calculated by Equation (9) under the assumption of γ_0_ = γ for wet-brush behavior and the equation for dry-brush blend. The calculated *D*/*D*_0_ of dry-brush blend was always much larger than the observed values under a given composition, confirming that the blends studied here did not show dry-brush behavior. The model predicted monotonic increase of *D*/*D*_0_ with increasing h-P4VP composition for wet-brush behavior. The composition variation of *D*/*D*_0_ of EO4VP/h-P4VP4 blend agreed quite well with the calculated result, as did the corresponding result of *a_j_*/*a*_*j*0_ (see Figure 6), suggesting that the h-P4VP4 was uniformly solubilized in P4VP microdomains and its presence did not alter the surface free energy significantly. 

On the other hand, the observed composition dependences of *D*/*D*_0_ and *a_j_*/*a*_*j*0_ of the blends with lower homopolymer molecular weights deviated obviously from the theoretical predictions, where the observed junction point separations were always larger than the calculated values (see Figure 6). Consequently, an excess swelling of the junction point separation occurred in the blends with sufficiently low homopolymer molecular weight (< ca. 4000 g/mol). The comparison was relative to the junction point separation calculated by assuming that the surface free energy was unperturbed after blending, i.e., *γ* = *γ_0_*. According to Equation (8), *a_j_* becomes larger when *γ* < *γ_0_*; therefore, the excess swelling of *a_j_* was attributed to the decrease of surface free energy under the presence of h-P4VP in the microdomain. 

**Figure 7:**
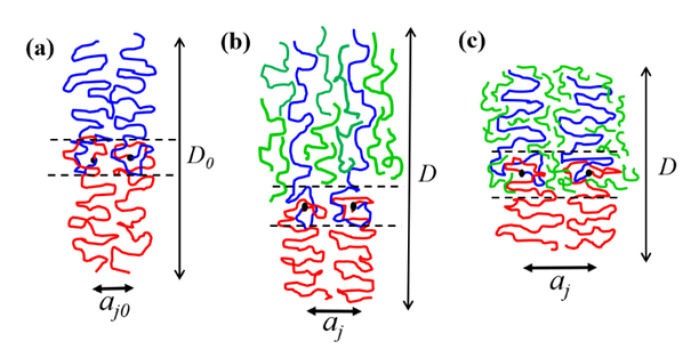
Schematic illustration of the structure of the interface in EO4VP and EO4VP/h-P4VP blends: (**a**) neat EO4VP in which the interface composes EO and 4VP segments from the constituent blocks; (**b**) EO4VP/h-P4VP blend with higher *M_h-P4VP_*, in which the h-P4VP chains were segregated out of the interfacial region completely; (**c**) EO4VP/h-P4VP blend with low *M_h-P4VP_*, in which a fraction of h-P4VP chains or their segments entered the interfacial region. The polymer chains in blue, red and green color represent the P4VP block, PEO block and h-P4VP, respectively.

The interface between the PEO and P4VP lamellar domains was formed by the gradient mixing of EO and 4VP segments, as schematically illustrated in Figure 7a, and the surface free energy composes both enthalpic and entropic components, i.e., *γ* = *γ_H_* + *γ_S_*. γ_H_ is related to the interaction energy between EO and 4VP segments (i.e., *γ_H_* ~ *χ*^1/2^); *γ_S_* consists of the contributions from (a) the gain of entropy from the combinatorial mixing of dissimilar segments, (b) the loss of entropy arising from the confinement of A (B) blocks in the interface, and (c) the loss of entropy stemming from the reduction of free volume of EO segments upon mixing with 4VP segments in the interface [24]. These three entropic terms are denoted as *γ_S,mix_*, *γ_S,conf_* and *γ_S,fv_*, respectively; *γ_S,fv_* is a term that is particularly significant in the LCOT system. 

We propose that the solubilization of h-P4VP in the microdomain may effectively reduce *γ_S,fv_*, which in turn caused the excess swelling of the junction point separation. When h-P4VP was solubilized in P4VP microdomain, there were two ways to establish the interfacial composition profile. In the first scenario, h-P4VP chains were segregated out of the interfacial region completely, such that the interface composed the mixture of the segments associated with PEO and P4VP block chains, as schematically illustrated in Figure 7b. In this case, the surface free energy of the blend should approximately equal that of the neat copolymer, i.e., *γ* ≈ *γ_0_*. This was likely the case for the h-P4VP4 blend, where the composition variation of *a_j_*/*a*_*j*0_ agreed well with that predicted by Equation (8) assuming unperturbed surface free energy. 

In the second scenario, as schematically illustrated in Figure 7c, a fraction of h-P4VP chains or their segments entered the interfacial region to replace some segments of P4VP block chains in establishing the composition profile in the interface. The interface then composed the segments of PEO blocks, P4VP blocks and h-P4VP chains. The replacement of P4VP block segments by the homopolymer segments may likely alleviate the free volume constraint imposed on PEO, because the short h-P4VP chains have higher free volume than the longer P4VP blocks due to the absence of junction point constraint and lower molecular weight. In this case, EO segments might have a higher free volume if they mix with h-P4VP chains. The alleviation of the free volume constraint reduced *γ_S,f_*_v_, and hence allowed a greater swelling of the junction point separation.

## 4. Conclusions

We demonstrated that a lamellae-forming PEO-*b*-P4VP and its blends with h-P4VP showed the feature of LCOT phase behavior, where the segregation strength reduced with decreasing temperature. Over the range of *r* value studied (*r* = 0.14–0.60), h-P4VP was found to solubilize uniformly in the P4VP microdomain, indicating that the wet-brush criterion established for the conventional UCOT system still applied to the LCOT blend. Nevertheless, when h-P4VP molecular weight was sufficiently low, excess swelling of the junction point separation was observed as compared to the theoretical prediction assuming unperturbed surface free energy. This excess swelling, which became more evident at lower homopolymer molecular weight, even led to a decrease of interdomain distance with increasing h-P4VP composition. The large junction point separation was attributed to the reduction of the surface free energy upon incorporating h-P4VP into the P4VP microdomain. We proposed that, when the h-P4VP molecular weight was sufficiently low, the homopolymer chains could enter the interfacial region to replace a portion of 4VP segments from P4VP block to mix with EO segments for establishing the composition gradient in the interface. In this case, the constraint of free volume of EO arising from the mixing with 4VP segments was alleviated, because short h-P4VP chain had a larger free volume than P4VP block. The contribution of the entropic penalty associated with the free volume to the surface free energy was thus reduced, thereby allowing a larger degree of swelling of the junction point separation to relieve the stretching of the P4VP blocks and h-P4VP chains in the microdomain.

## Figures and Tables

**Table 1:** The molecular characteristics of the copolymer and homopolymer samples used in this study.

Sample ID	Sample	*M*n (g/mol)	*M*_n,PEO_ (g/mol)	*M*_n,P4VP_ (g/mol)	*M*_w_/*M*_n_	*f_4VP_* ^1^
EO4VP	PEO-*b*-P4VP	12200	5000	7200	1.28	0.61
h-P4VP1	P4VP	1000	-	1000	1.20	-
h-P4VP2	P4VP	1700	-	1700	1.19	-
h-P4VP3	P4VP	2300	-	2300	1.15	-
h-P4VP4	P4VP	4300	-	4300	1.20	-

^1^ volume fraction of P4VP block in the diblock copolymer.

## Supplementary Materials

The following are available online at https://www.mdpi.com/article/10.3390/polym13193415/s1, **Table S1**: The compositions of the EO4VP/h-P4VP blends prepared in this study. **Figure S1**: (**a**) The temperature dependence of the specific volumes of P4VP and PEO homopolymer at 1 bar obtained by the Tait model; (**b**) The electron densities of PEO and P4VP as a function of temperature calculated from the specific volume data in (**a**). **Figure S2**: Temperature-dependent SAXS profiles of the PEO-b-P4VP/h-P4VP blend with the overall volume fraction of P4VP of 0.68 collected in a cooling cycle (**a**) from 180 to 120 °C, (**b**) from 110 to 90 °C and (**c**) from 75 to 45 °C.
