# Supplementary material for: Solubilization Behavior of Homopolymer in Its Blend with the Block Copolymer Displaying the Feature of Lower Critical Ordering Transition
Source: Polymers (Basel). 2021 Oct 5;13(19):3415. doi: 10.3390/polym13193415 (PMC8512391; doi:10.3390/polym13193415)
Supplement: Supplementary file 1 [file polymers-13-03415-s001.zip › polymers-1407358-supplementary.pdf]

Supporting Information

Solubilization Behavior of Homopolymer in Its Blend with the Block Copolymer Displaying the Feature of Lower Critical Ordering Transition

Yu-Hsuan Lin[1], Chang-Cheng Shiu[1], Tien-Lin Chen[2], Hsin-Lung Chen[1*], and Jing-Cherng Tsai[2]

[1]Department of Chemical Engineering, National Tsing Hua University, Hsinchu 30013, Taiwan

[2]Department of Chemical Engineering, National Chung Cheng University, Chia-Yi 62102, Taiwan

1. The compositions of the EO4VP/h-P4VP blends prepared in this study.

Table S1. The compositions of the EO4VP/h-P4VP blends prepared in this study.

Sample	f_{P4VP}	Φ_{EO4VP}
EO4VP	0.61	1.00
EO4VP/h-P4VP1 blend	0.66	0.85
	0.69	0.78
	0.71	0.73
EO4VP/h-P4VP2 blend	0.66	0.85
	0.69	0.78
	0.71	0.73
EO4VP/h-P4VP3 blend	0.66	0.85
	0.69	0.78
	0.71	0.73
EO4VP/h-P4VP4 blend	0.66	0.85
	0.69	0.78
	0.71	0.73

*f_{P4VP} is the total volume fraction of P4VP and Φ_{EO4VP} signifies the volume fraction of the deblock copolymer in the blend.

2.The temperature dependence of the electron densities of PEO and P4VP

The specific volumes of PEO and P4VP were measured at different temperatures by a piston type PVT instrument (GOTECH PVT-6000). Isobaric cooling procedure with the cooling rate 5 ℃/min was adopted to obtain that the specific volume as a function of temperature in a cooling process. The testing procedure was standardized in ISO 17744:2004. The specific volume measurement was conducted at different pressures greater than the atmospheric pressure. The PVT data of the samples at the atmospheric pressure were obtained by Tait model fitting. For an isothermal compressibility model (i.e., a volume-pressure relationship), Tait equation is given by[1-2]

$$v(T,P)=v_0(T)\left\{1-C\ln\left[1+\frac{P}{B(T)}\right]\right\}+v_t(T,P)$$

The Tait model fitting results can be obtained by adjusting several parameters to satisfy least squares estimation.

Figure S1(a) shows the measured specific volumes of PEO and P4VP homopolymers ($M_{n,PEO}$ = 10000 g/mol; $M_{n,P4VP}$ = 7000 g/mol) as a function of temperature. The disparity in thermal expansion coefficients was manifested by the different slopes in the plot, where the specific volume of PEO increased more rapidly than that of P4VP with increasing temperature. The thermal expansion coefficient of PEO was found to be 7.55×10^{-4} K^{-1}. In the case of P4VP, the change of slope at ca. 100 ºC was due to glass transition; the thermal expansion coefficients in the glassy and rubbery state were 8.5×10^{-5} and 5×10^{-4} K^{-1}, respectively.

The measured specific volumes were used to calculate the electron densities of the two polymers as a function of temperature, as displayed in Figure S1(b). It can be seen that the electron density contrast given by the difference in electron densities between P4VP and PEO decreased with decreasing temperature. The match point was found to locate at about 60 ºC; however, the scattering peaks of the lamellar structure did not vanish completely at 60 ºC (see Figure 1 of the paper). The discrepancy can be explained by the fact that the cooling rate adopted in the dilatometry experiment was faster than that associated with the SAXS measurement, as the dilatometry measurement from

100 to 60 °C took five minutes to complete, while it took more than 300 minutes in the SAXS measurement, because the sample was equilibrated at each temperature for 30 minutes. As a result, the nonequilibrium glassy domain of P4VP had more time to relax in the SAXS experiment, leading to higher mass density (and hence electron density contrast) than that attained in the dilatometry measurement.

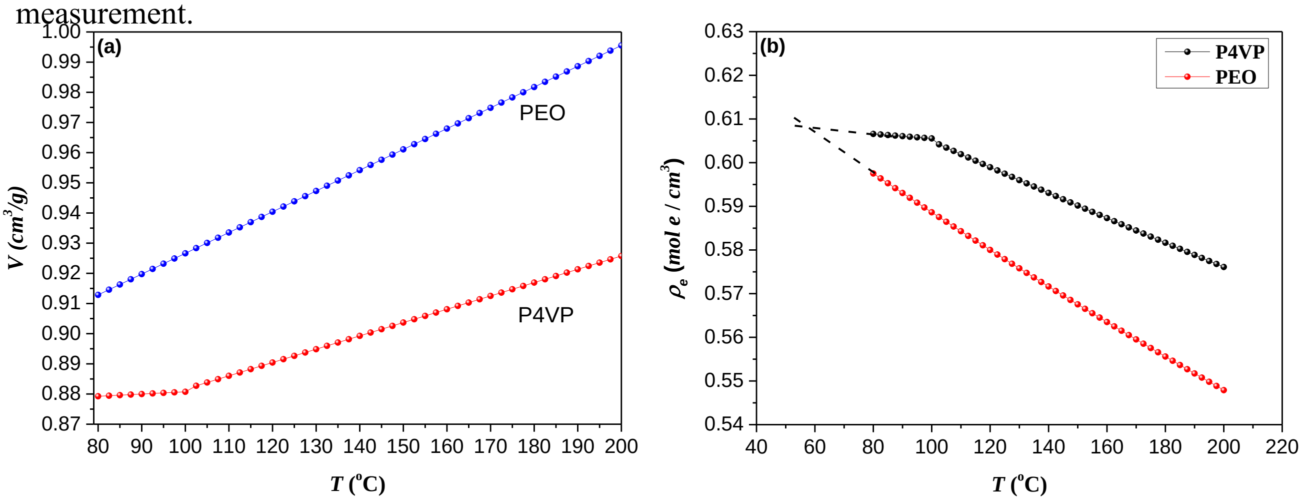

Figure S1. (a) The temperature dependence of the specific volumes of P4VP and PEO homopolymer at 1 bar obtained by the Tait model. **(b)** The electron densities of PEO and P4VP as a function of temperature calculated from the specific volume data in **(a)**.

3.Order-order transition from lamellar (*LAM*) structure to hexagonally-packed cylinder (*HEX*) morphology in a blend of PEO-*b*-P4VP with P4VP homopolymer in the cooling process.

The poly(ethylene oxide)-*block*-poly(4-vinylpyridine) (PEO-*b*-P4VP) with the molecular weight of $M_{\text{b-PEO}}$ = 4000 g/mol and $M_{\text{b-P4VP}}$ = 6000 g/mol (PDI＝1.16) was blended with a P4VP homopolymer with molecular weight of $M_{\text{h-P4VP}}$ = 1000 g/mol (PDI=1.20) to yield the blend with the overall P4VP volume fraction of 0.68. Figure S2 shows the temperauture-dependent SAXS profiles of the blend collected in a cooling cycle. The SAXS profile at 180 °C displayed two peaks with the position ratio of 1:2, signifying the formation of *LAM* morphology with D = 18.6 nm in the blend. As can be seen from enlarged SAXS profiles in Figure S2(a), the lamellar structure persisted from 180 to 120 °C in the cooling process. As the temperature was lowered to 110 °C, a small scattering peak

with the position of $(3)^{1/2}q_1$ (with q_1 being the position of the primary peak) was identified, as shown in Figure S2(b). This peak grew progressively on further cooling, as demonstrated in Figure S2(c). At 45 and 60 ºC, three diffraction peaks with the position ratio of 1 : $(3)^{1/2}$: 2 were clearly discernible, indicating the formation of *HEX* morphology in the blend. On basis of the temperature-dependent SAXS result, an order-order transition from *LAM* to *HEX* in the cooling process was revealed. This direction of phase transition was opposite to that displayed by the conventional UCOT diblocks; it hence offers a solid evidence for the decrease of segregation strength with decreasing temperature for PEO-*b*-P4VP system.

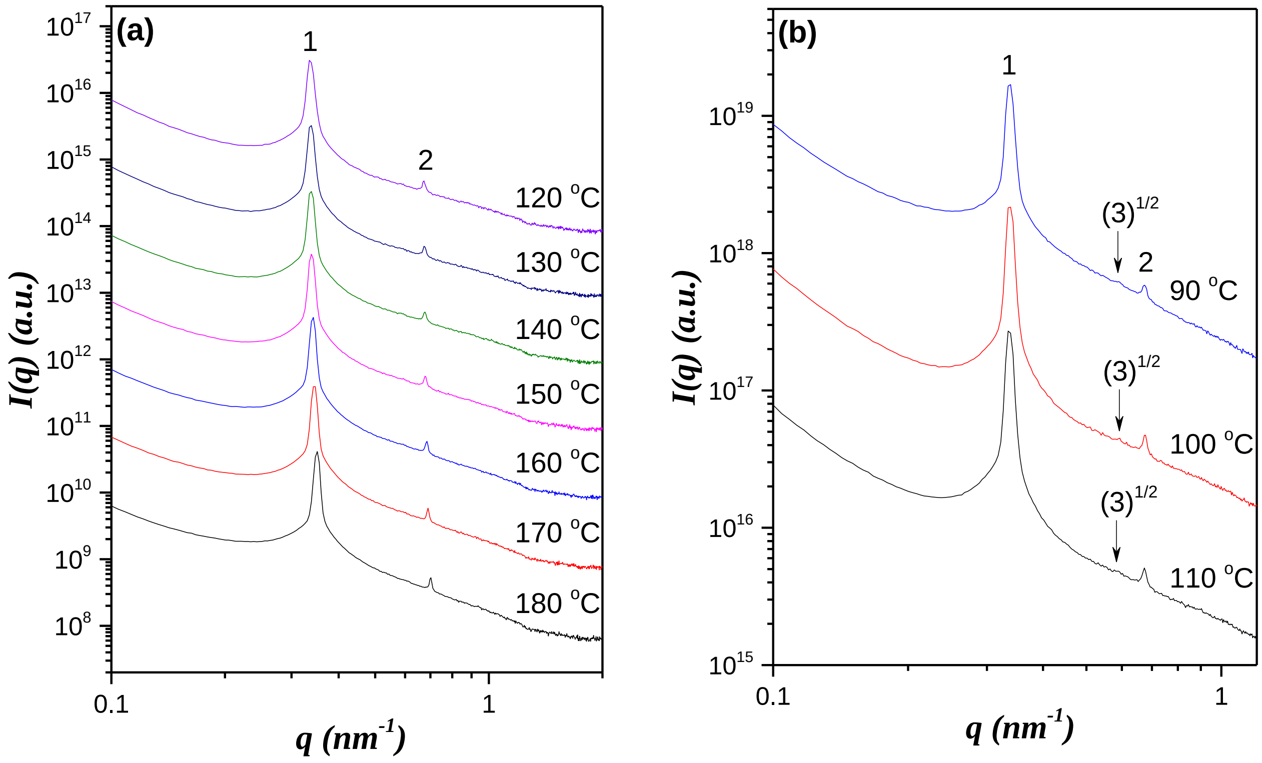

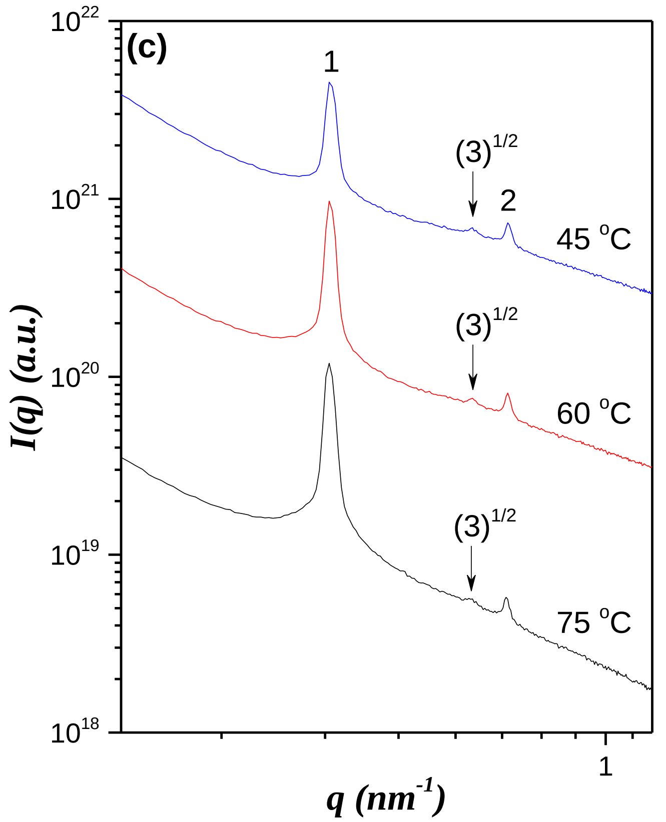

Figure S2. Temperature-dependent SAXS profiles of the PEO-*b*-P4VP/h-P4VP blend with the overall volume fraction of P4VP of 0.68 collected in a cooling cycle **(a)** from 180 to 120 °C, **(b)** from 110 to 90 °C and **(c)** from 75 to 45 °C.

References

1. Rodgers, P. A. Pressure–volume–temperature relationships for polymeric liquids: A review of equations of state and their characteristic parameters for 56 polymers. *Journal of Applied Polymer Science* **1993,** 48 (6), 1061-1080.

2. Zoller, P.; Fakhreddine, Y. A. Pressure—volume—temperature studies of semi-crystalline polymers. *Thermochimica acta* **1994,** 238, 397-415.
